# Supplementary material for: Application of Whole Exome Sequencing and Functional Annotations to Identify Genetic Variants Associated with Marfan Syndrome
Source: J Pers Med. 2022 Feb 1;12(2):198. doi: 10.3390/jpm12020198 (PMC8878617; doi:10.3390/jpm12020198)
Supplement: Supplementary file 1 [file jpm-12-00198-s001.zip › jpm-1462170-supplementary.pdf]

Supplementary Table S1. The list of previously reported MFS-related genes.

No	Gene Name	Location	Exons	Description	Reference
1	ACE	17q23.3	26	Angiotensin I Converting Enzyme	[1]
2	ACTA2	10q23.31	10	Actin Alpha 2, Smooth Muscle	[2, 3]
3	AGT	1q42.2	5	Angiotensinogen	[1]
4	COL1A1	17q21.33	51	Collagen Type I Alpha 1 Chain	[2, 4]
5	COL1A2	7q21.3	52	Collagen Type I Alpha 2 Chain	[2, 5]
6	COL3A1	2q32.2	51	Collagen Type III Alpha 1 Chain	[2]
7	COL5A1	9q34.3	67	Collagen Type V Alpha 1 Chain	[2, 4, 6]
8	COL5A2	2q32.2	55	Collagen Type V Alpha 2 Chain	[2, 4]
9	DSC2	18q12.1	17	Desmocollin 2	[7]
10	FBN1	15q21.1	65	Fibrillin 1	[2, 3, 7-9]
11	FBN2	5q23.3	65	Fibrillin 2	[9]
12	FBN3	19p13.2	68	Fibrillin 3	[10]
13	FLNA	Xq28	48	Filamin A	[2, 11]
14	LOX	5q23.1	8	Lysyl Oxidase	[12, 13]
15	LOXL1	15q24.1	9	Lysyl Oxidase Like 1	[12]
16	LRP1	12q13.3	90	LDL Receptor Related Protein 1	[7]
17	LTBP3	11q13.1	29	Latent Transforming Growth Factor Beta Binding Protein 3	[14]
18	MTHFR	1p36.22	12	Methylenetetrahydrofolate Reductase	[15]
19	MYH11	16p13.11	43	Myosin Heavy Chain 11	[2, 16]
20	MYLK	3q21.1	39	Myosin Light Chain Kinase	[2, 16]
21	NOTCH1	9q34.3	34	Notch Receptor 1	[2]
22	SMAD2	18q21.1	18	SMAD Family Member 2	[17]
23	SMAD3	15q22.33	13	SMAD Family Member 3	[2, 8]
24	SMAD4	18q21.2	12	SMAD Family Member 4	[2, 18]
25	TGFBR1	9q22.33	11	Transforming Growth Factor Beta Receptor 1	[2, 3, 8]
26	TGFBR2	3p24.1	11	Transforming Growth Factor Beta Receptor 2	[2, 3, 8]

Supplementary Table S2. Profile of 19 rare variants on MFS-related genes.

Chr.	Position	Alt.	Gene	Type	ClinVar Annotation		Prediction scoring	
					Disease	Significance	CADD	REVEL
1	11794020	A	MTHFR	nsSNV	-	-	26.4	0.21
2	189051324	C	COL5A2	nsSNV	-	-	22.8	0.368
3	30623240	C	TGFBR2	nsSNV	Thoracic aortic aneurysm and aortic dissection	Likely benign	6.806	0.188
9	99105216	T	TGFBR1	nsSNV	-	-	24.5	0.259
**9**	**134758250**	**A**	**COL5A1**	**nsSNV**	**Ehlers-Danlos syndrome, classic type**	**Conflicting interpretations of pathogenicity**	**27**	**0.674**
11	65557857	CCAG CAGC AG,C	LTBP3	Ins (NF)	-	-	-	-
12	57167041	T	LRP1	nsSNV	-	-	24.4	0.611
15	48420690	T	FBN1	nsSNV	-	Uncertain significance	23.9	0.377
**15**	**48436991**	**A**	**FBN1**	**nsSNV**	**-**	**-**	**34**	**0.941**
15	48437824	T	FBN1	nsSNV	-	-	24	0.525
**15**	**48448860**	**T**	**FBN1**	**nsSNV**	**Marfan syndrome, Familial thoracic aortic aneurysms and dissections**	**Pathogenic**	**32**	**0.988**
15	48487321	G	FBN1	Del (F)	-	-	-	-
**15**	**48503803**	**G**	**FBN1**	**Del (F)**	**Marfan syndrome, Loeys-dietz syndrome, Familial thoracic aortic aneurysms and dissections**	**Likely pathogenic**	**-**	**-**
**16**	**15714999**	**C**	**MYH11**	**nsSNV**	**Aortic aneurysm, familial thoracic 4**	**Conflicting interpretations of pathogenicity**	**10.06**	**0.266**
17	63494011	T	ACE	nsSNV	-	-	29	0.465
17	63497343	T	ACE	stop gain	-	-	45	-
19	8123973	T	FBN3	nsSNV	-	-	23.1	0.587
19	8133099	C	FBN3	Del (NF)	-	-	-	-
**19**	**8136499**	**G**	**FBN3**	**nsSNV**	**-**	**-**	**24.5**	**0.83**

Pathogenic variants are shown in bold

Supplementary Table S3. Systemic score of MFS patients.

Feature	Patients									
	**1**	**2**	**3**	**4**	**5**	**6**	**7**	**8**	**9**	**11**
Wrist and thumb =3 (weist or thumb sign=1)	1	0	0	1	1	1	0	3	0	1
Pectus carinatum deformity = 2 (pectus excavatum or chest asymmetry = 1)	2	1	0	2	1	0	0	1	0	2
Hindfoot deformity = 2 (plain pes planus = 1)	1	1	0	0	0	1	1	0	1	2
Dural extasia = 2	2	0	0	2	0	0	0	0	0	0
Protrusio acetabuli = 2	0	0	0	0	2	0	2	0	0	0
pneumothroax = 2	0	0	0	0	0	0	0	0	2	0
Reduced upper segement/lower segment ration and increased arm/height and no severe scoliosis = 1	0	0	0	0	0	0	0	0	0	0
Scoliosis or thoracolumbar kyphosis = 1	1	1	1	0	0	0	1	1	1	1
Reduced elbow extension = 1	0	0	0	0	0	0	0	0	0	0
Mitral valve prolapse = 1	1	1	1	0	1	1	0	0	0	0
Facial features (3/5) = 1 (dolichocephaly, enophthalmos, downslanting palpebral fissures, malar hypoplasia, retrognathia)	0	0	0	0	0	0	0	0	0	0
Skin striae (stretch marks) = 1	1	0	1	1	1	0	0	0	0	0
Myopia > 3 diopters = 1	0	1	1	1	1	1	0	1	0	1
Systemic score	9	5	4	7	7	4	4	6	4	7

Supplementary Table S4. MFS candidate genes.

Human Associated Gene Name (N = 219)	Mouse Associated Gene Name (N = 229)	Ensembl gene ID (N = 230)	Note
ABCA12	Abca12	ENSMUSG00000050296	-
ABCC12	Abcc12	ENSMUSG00000036872	-
ABCC6	Abcc6	ENSMUSG00000030834	-
ABCC9	Abcc9	ENSMUSG00000030249	-
ACACB	Acacb	ENSMUSG00000042010	-
ACE	Ace	ENSMUSG00000020681	-
ADGRG1	Adgrg1	ENSMUSG00000031785	-
AGL	Agl	ENSMUSG00000033400	-
AGXT	Agxt	ENSMUSG00000026272	-
AKAP9	Akap9	ENSMUSG00000040407	-
ALAS1	Alas1	ENSMUSG00000032786	-
ALG13	Alg13	ENSMUSG00000041718	-
ALX1	-	-	Can't mapped to mouse gene name
ANKRD33B	Ankrd33b	ENSMUSG00000022237	-
AREL1	Arel1	ENSMUSG00000042350	-
ARFGAP3	Arfgap3	ENSMUSG00000054277	-
ASS1	Ass1	ENSMUSG00000076441	-
ATAD3A	Atad3a	ENSMUSG00000029036	-
ATL2	Atl2	ENSMUSG00000059811	-
ATP10B	Atp10b	ENSMUSG00000055415	-
ATP11B	Atp11b	ENSMUSG00000037400	-
ATP2B1	Atp2b1	ENSMUSG00000019943	-
ATP6V0A1	Atp6v0a1	ENSMUSG00000019302	-
ATP6V1B1	Atp6v1b1	ENSMUSG00000006269	-
ATP7B	Atp7b	ENSMUSG00000006567	-
ATXN1	Atxn1	ENSMUSG00000046876	-
B3GLCT	B3glct	ENSMUSG00000051950	-
BAG4	Bag4	ENSMUSG00000037316	-
BARD1	Bard1	ENSMUSG00000026196	-
BBX	Bbx	ENSMUSG00000022641	-
BCKDHA	Bckdha	ENSMUSG00000060376	-
BUB1	Bub1	ENSMUSG00000027379	-
CACNA1C	Cacna1c	ENSMUSG00000051331	-
CAVIN2	Cavin2	ENSMUSG00000045954	-
CAVIN4	Cavin4	ENSMUSG00000028348	-
CDC40	Cdc40	ENSMUSG00000038446	-
CDH23	Cdh23	ENSMUSG00000012819	-
CDH4	Cdh4	ENSMUSG00000000305	-
CELSR3	Celsr3	ENSMUSG00000023473	-
CEP120	Cep120	ENSMUSG00000048799	-
CEP290	Cep290	ENSMUSG00000019971	-
CES2	Ces2b	ENSMUSG00000062826	Mapped to multiple mouse gene name
CES2	Ces2f	ENSMUSG00000061825	Mapped to multiple mouse gene name
CES2	Ces2a	ENSMUSG00000055730	Mapped to multiple mouse gene name
CES2	Ces2e	ENSMUSG00000031877	Mapped to multiple mouse gene name
CES2	Ces2c	ENSMUSG00000031886	Mapped to multiple mouse gene name
CES2	Ces2g	ENSMUSG00000050097	Mapped to multiple mouse gene name
CFAP65	Cfap65	ENSMUSG00000047021	-
CFD	Cfd	ENSMUSG00000061780	-
CFL2	Cfl2	ENSMUSG00000062929	-
CHD3	Chd3	ENSMUSG00000018474	-
CHKB	Gm44502	ENSMUSG00000022617	Mapped to multiple mouse gene name
CHKB	Chkb	ENSMUSG00000116461	Mapped to multiple mouse gene name
CHRNA4	Chrna4	ENSMUSG00000027577	-
CIDEB	Cideb	ENSMUSG00000022219	-
CNGB3	Cngb3	ENSMUSG00000056494	-

COL4A3	Col4a3	ENSMUSG00000079465	-
COL5A1	Col5a1	ENSMUSG00000026837	-
COL5A2	Col5a2	ENSMUSG00000026042	-
COL6A3	Col6a3	ENSMUSG00000048126	-
COL6A6	Col6a6	ENSMUSG00000043719	-
CORIN	Corin	ENSMUSG00000005220	-
CPLANE1	Cplane1	ENSMUSG00000039801	-
CRISPLD1	Crispld1	ENSMUSG00000025776	-
CSK	Csk	ENSMUSG00000032312	-
CTSA	Ctsa	ENSMUSG00000017760	-
CUBN	Cubn	ENSMUSG00000026726	-
CYB5R2	Cyb5r2	ENSMUSG00000048065	-
CYP11B1	-	-	Can't mapped to mouse gene name
CYP2J2	Cyp2j9	ENSMUSG00000028571	Mapped to multiple mouse gene name
CYP2J2	Cyp2j13	ENSMUSG00000015224	Mapped to multiple mouse gene name
CYP4F3	Cyp4f18	ENSMUSG00000003484	-
DDOST	Ddost	ENSMUSG00000028757	-
DHCR24	Dhcr24	ENSMUSG00000034926	-
DNAH7	Dnah7a	ENSMUSG00000096141	Mapped to multiple mouse gene name
DNAH7	Dnah7c	ENSMUSG00000101337	Mapped to multiple mouse gene name
DNAH7	Dnah7b	ENSMUSG00000041144	Mapped to multiple mouse gene name
DNAH8	Dnah8	ENSMUSG00000033826	-
DSG2	-	-	Can't mapped to mouse gene name
EARS2	Ears2	ENSMUSG00000030871	-
EBAG9	Ebag9	ENSMUSG00000022339	-
EIPR1	Eipr1	ENSMUSG00000036613	-
EP400	Ep400	ENSMUSG00000029505	-
EPC2	Epc2	ENSMUSG00000069495	-
ESPNL	Espnl	ENSMUSG00000049515	-
ESRRG	Esrrg	ENSMUSG00000026610	-
EXOC5	Exoc5	ENSMUSG00000061244	-
FAM126A	Fam126a	ENSMUSG00000028995	-
FAS	Fas	ENSMUSG00000024778	-
FAT1	Fat1	ENSMUSG00000070047	-
FBN1	Fbn1	ENSMUSG00000027204	-
FBN3	-	-	Can't mapped to mouse gene name
FBXO16	Fbxo16	ENSMUSG00000034532	-
FLG	Flg	ENSMUSG00000102439	-
FLNC	Flnc	ENSMUSG00000068699	-
FMO3	Fmo3	ENSMUSG00000026691	-
FRY	Fry	ENSMUSG00000056602	-
GARNL3	Garnl3	ENSMUSG00000038860	-
GC	Gc	ENSMUSG00000035540	-
GFM1	Gfm1	ENSMUSG00000027774	-
GIPC2	Gipc2	ENSMUSG00000039131	-
GLRA1	Glra1	ENSMUSG00000000263	-
GPD2	Gpd2	ENSMUSG00000026827	-
GPRC5C	Gprc5c	ENSMUSG00000051043	-
GRK7	-	-	Can't mapped to mouse gene name
GSS	Gss	ENSMUSG00000027610	-
HABP2	Habp2	ENSMUSG00000025075	-
HCFC1	Hcfc1	ENSMUSG00000031386	-
HGSNAT	Hgsnat	ENSMUSG00000037260	-
IL17RD	Il17rd	ENSMUSG00000040717	-
ITGA6	Itga6	ENSMUSG00000027111	-
KATNAL1	Katnal1	ENSMUSG00000041298	-
KDM5B	Kdm5b	ENSMUSG00000042207	-
KIF9	Kif9	ENSMUSG00000032489	-
KLHL40	Klhl40	ENSMUSG00000074001	-
KRT82	Krt82	ENSMUSG00000049548	-
L2HGDH	L2hgdh	ENSMUSG00000020988	-

LAMA3	Lama3	ENSMUSG00000024421	-
LAMC3	Lamc3	ENSMUSG00000026840	-
LDB3	Ldb3	ENSMUSG00000021798	-
LDLR	Ldlr	ENSMUSG00000032193	-
LIMA1	Lima1	ENSMUSG00000023022	-
LPIN1	Lpin1	ENSMUSG00000020593	-
LRCH4	Lrch4	ENSMUSG00000029720	Mapped to multiple mouse gene name
LRCH4	Gm20605	ENSMUSG00000093445	Mapped to multiple mouse gene name
LRP1	Lrp1	ENSMUSG00000040249	-
LRP1B	Lrp1b	ENSMUSG00000049252	-
LRP4	Lrp4	ENSMUSG00000027253	-
LTBP3	Ltbp3	ENSMUSG00000024940	-
LTK	Ltk	ENSMUSG00000027297	-
MAT2A	Mat2a	ENSMUSG00000053907	-
MATN4	Matn4	ENSMUSG00000016995	-
MCCC2	Mccc2	ENSMUSG00000021646	-
ME2	Me2	ENSMUSG00000024556	-
MEGF10	Megf10	ENSMUSG00000024593	-
MFN1	Mfn1	ENSMUSG00000027668	-
MPI	Mpi	ENSMUSG00000032306	-
MSH2	Msh2	ENSMUSG00000024151	-
MTHFR	Mthfr	ENSMUSG00000029009	-
MUSK	Musk	ENSMUSG00000057280	-
MYBPC3	Mybpc3	ENSMUSG00000002100	-
MYH11	Myh11	ENSMUSG00000018830	-
MYH8	Myh8	ENSMUSG00000055775	-
MYO1H	Myo1h	ENSMUSG00000066952	-
MYO5C	Myo5c	ENSMUSG00000033590	-
MYO9A	Myo9a	ENSMUSG00000039585	-
MYOT	Myot	ENSMUSG00000024471	-
NAPA	Napa	ENSMUSG00000006024	-
NCF1	Ncf1	ENSMUSG00000015950	-
NEK3	Nek3	ENSMUSG00000031478	-
NELL2	Nell2	ENSMUSG00000022454	-
NHLRC1	Nhlrc1	ENSMUSG00000044231	-
NOTCH3	Notch3	ENSMUSG00000038146	-
NPC1	Npc1	ENSMUSG00000024413	-
NPL	Npl	ENSMUSG00000042684	-
NR0B2	Nr0b2	ENSMUSG00000037583	-
NRCAM	Nrcam	ENSMUSG00000020598	-
NRXN1	Nrxn1	ENSMUSG00000024109	-
OPA1	Opa1	ENSMUSG00000038084	-
OTOF	Otof	ENSMUSG00000062372	-
PANK2	Pank2	ENSMUSG00000037514	-
PAQR6	Paqr6	ENSMUSG00000041423	-
PAX6	Pax6	ENSMUSG00000027168	-
PCCA	Pcca	ENSMUSG00000041650	-
PCSK9	Pcsk9	ENSMUSG00000044254	-
PDE6A	Pde6a	ENSMUSG00000024575	-
PGC	Pgc	ENSMUSG00000023987	-
PICK1	Pick1	ENSMUSG00000068206	Mapped to multiple mouse gene name
PICK1	Gm49486	ENSMUSG00000116121	Mapped to multiple mouse gene name
PIGW	Pigw	ENSMUSG00000045140	-
PKHD1	Pkhd1	ENSMUSG00000043760	-
PKP2	Pkp2	ENSMUSG00000041957	-
PLAT	Plat	ENSMUSG00000031538	-
PLCB2	Plcb2	ENSMUSG00000040061	-
PNPLA7	Pnpla7	ENSMUSG00000036833	-
PNPLA8	Pnpla8	ENSMUSG00000036257	-
POLG	Polg	ENSMUSG00000039176	-
POMT1	Pomt1	ENSMUSG00000039254	-

POU4F3	Pou4f3	ENSMUSG00000024497	-
PPTC7	Pptc7	ENSMUSG00000038582	-
PRKCSH	Prkcsh	ENSMUSG00000003402	-
PRKRA	Prkra	ENSMUSG00000002731	-
PRSS1	Prss2	ENSMUSG00000071521	Mapped to multiple mouse gene name
PRSS1	Gm10334	ENSMUSG00000036938	Mapped to multiple mouse gene name
PRSS1	Prss3	ENSMUSG00000054106	Mapped to multiple mouse gene name
PRSS1	Prss1	ENSMUSG00000071517	Mapped to multiple mouse gene name
PRSS1	Gm5771	ENSMUSG00000071519	Mapped to multiple mouse gene name
PRSS1	Try4	ENSMUSG00000058119	Mapped to multiple mouse gene name
PRSS1	Try10	ENSMUSG00000062751	Mapped to multiple mouse gene name
PRSS1	Try5	ENSMUSG00000057163	Mapped to multiple mouse gene name
PTEN	Pten	ENSMUSG00000013663	-
PTPRR	Ptprr	ENSMUSG00000020151	-
RAB3GAP2	Rab3gap2	ENSMUSG00000039318	-
RBM20	Rbm20	ENSMUSG00000043639	-
RNF175	-	-	Can't mapped to mouse gene name
RPS6KA6	Rps6ka6	ENSMUSG00000025665	-
SACM1L	Sacm1l	ENSMUSG00000025240	-
SARDH	Sardh	ENSMUSG00000009614	-
SCN4B	Scn4b	ENSMUSG00000046480	-
SDHA	Sdha	ENSMUSG00000021577	-
SECISBP2L	Secisbp2l	ENSMUSG00000035093	-
SHPRH	Shprh	ENSMUSG00000090112	-
SLC16A1	Slc16a1	ENSMUSG00000032902	-
SLC16A14	Slc16a14	ENSMUSG00000026220	-
SLC22A1	Slc22a1	ENSMUSG00000023829	-
SLC22A4	Slc22a4	ENSMUSG00000020334	-
SLC25A5	Slc25a5	ENSMUSG00000016319	-
SLC35G6	Slc35g3	ENSMUSG00000018776	-
SMCHD1	Smchd1	ENSMUSG00000024054	-
SMOX	Smox	ENSMUSG00000027333	-
SNX27	Snx27	ENSMUSG00000028136	-
SOD3	Sod3	ENSMUSG00000072941	-
SOX10	Sox10	ENSMUSG00000033006	-
SPATA5	Spata5	ENSMUSG00000027722	-
SPATA7	Spata7	ENSMUSG00000021007	-
SPINT1	Spint1	ENSMUSG00000027315	-
SSBP2	Ssbp2	ENSMUSG00000003992	-
STX10	-	-	Can't mapped to mouse gene name
TBX3	Tbx3	ENSMUSG00000018604	-
TCTN2	-	ENSMUSG00000118662	Can't mapped to mouse gene name
TGFBR1	Tgfbr1	ENSMUSG00000007613	-
TGFBR2	Tgfbr2	ENSMUSG00000032440	-
TMEM82	Tmem82	ENSMUSG00000043085	-
TRIM17	Trim17	ENSMUSG00000036964	-
TRIP13	Trip13	ENSMUSG00000021569	-
TRRAP	Trrap	ENSMUSG00000045482	-
TTI2	Tti2	ENSMUSG00000031577	-
TTN	Ttn	ENSMUSG00000051747	-
TYRP1	Tyrp1	ENSMUSG00000005994	-
UNC13C	Unc13c	ENSMUSG00000062151	-
UNC13D	Unc13d	ENSMUSG00000057948	-
USH2A	Ush2a	ENSMUSG00000026609	-
VWF	Vwf	ENSMUSG00000001930	-
WDR11	Wdr11	ENSMUSG00000042055	-
WHRN	Whrn	ENSMUSG00000039137	-
ZNF469	Zfp469	ENSMUSG00000043903	-
ZRANB3	Zranb3	ENSMUSG00000036086	-

Supplementary Table S5. Rare variants on MFS candidate genes carried by patients no.2.

Chr.	Position	Alt.	Gene	Type	TWB	Number of Alt. allele in MFS patients										
						1	2	3	4	5	6	7	8	9	11	10[a]
2	178573830	T	TTN	nsSNV	0.0035	-	1	-	-	-	-	-	-	-	-	-
2	240869010	C	AGXT	nsSNV	0.01	-	1	-	-	-	-	-	-	-	-	-
2	11815153	G	LPIN1	nsSNV	-	-	1	-	-	-	-	-	-	-	-	-
3	57106133	A	IL17RD	nsSNV	0.002	-	1	-	-	-	-	-	-	-	-	-
5	132335857	C	SLC22A4	nsSNV	0.002	-	1	-	-	-	-	-	-	-	-	-
5	146339918	G	POU4F3	nsSNV	0.0005	-	1	-	-	-	-	-	-	-	-	-
6	18121516	A	NHLRC1	nsSNV	0.0035	-	1	-	-	-	-	-	-	-	-	-
7	92110149	C	AKAP9	nsSNV	0.007	-	1	-	-	-	-	-	-	-	-	-
7	142752950	G	PRSS1	nsSNV	-	1	1	1	-	-	1	-	1	-	1	-
7	108191788	A	NRCAM	nsSNV	-	-	1	-	-	-	-	-	-	-	-	-
7	142750621	G	PRSS1	nsSNV	0.004	-	1	-	-	-	-	-	-	-	-	-
8	33500476	C	TTI2	nsSNV	0.008	-	1	-	-	-	-	-	-	-	-	-
8	42180344	C	PLAT	nsSNV	0.005	-	1	-	-	-	-	-	-	-	-	-
9	127335269	T	GARNL3	nsSNV	-	-	1	-	-	-	-	-	-	-	-	-
10	110812415	A	RBM20	nsSNV	0.0015	-	1	-	-	-	-	-	-	-	-	-
12	32843271	A	PKP2	nsSNV	0.002	-	1	-	-	-	-	-	-	-	-	-
16	23552164	C	EARS2	nsSNV	0.0035	-	1	-	-	-	-	-	-	-	-	-
16	48105194	G	ABCC12	nsSNV	0.0005	-	1	-	-	-	-	-	-	-	-	-
17	10406098	C	MYH8	nsSNV	-	-	1	-	-	-	-	-	-	-	-	-
17	74440560	C	GPRC5C	nsSNV	0.0005	-	1	-	-	-	-	-	-	-	-	-
20	3889380	G	PANK2	nsSNV	0.01	-	1	-	-	-	-	-	-	-	-	-
20	61936812	A	CDH4	nsSNV	0.001	-	1	-	-	-	-	-	-	-	-	-
22	42847628	A	ARFGAP3	nsSNV	-	-	1	-	-	-	-	-	-	-	-	-

[a]Healthy subject.

Supplementary Table S6. Rare variants on MFS candidate genes carried by patients no.5.

Chr.	Position	Alt.	Gene	Type	TWB	Number of Alt. allele in MFS patients										
						1	2	3	4	5	6	7	8	9	11	10[a]
1	171108182	C	FMO3	stopgain	-	-	-	-	-	1	-	-	-	-	-	-
2	47429786	G	MSH2	nsSNV	0.0015	-	-	-	-	1	-	-	-	-	-	-
2	214951036	C	ABCA12	nsSNV	0.003	-	-	-	-	1	-	-	-	-	-	-
3	141807906	T	GRK7	nsSNV	0.003	-	-	-	-	1	-	-	-	-	-	-
3	193642978	A	OPA1	nsSNV	-	-	-	-	-	1	-	-	-	-	-	-
4	186663613	A	FAT1	nsSNV	-	-	-	-	-	1	-	-	-	-	-	-
5	893075	G	TRIP13	nsSNV	0.001	-	-	-	-	1	-	-	-	-	-	-
5	236504	C	SDHA	nsSNV	-	-	-	-	-	1	-	-	-	-	-	-
5	151859872	C	GLRA1	nsSNV	-	-	-	-	-	1	-	-	-	-	-	-
7	74779296	A	NCF1	nsSNV	0.002	-	-	-	-	1	-	-	-	-	-	-
8	43173733	C	HGSNAT	nsSNV	-	-	-	-	-	1	-	-	-	-	-	-
9	100578538	A	CAVIN4	nsSNV	0.002	-	-	-	-	1	-	-	-	-	-	-
12	21829038	A	ABCC9	nsSNV	0.004	-	-	-	-	1	-	-	-	-	-	-
13	30210320	T	KATNAL1	nsSNV	0.0035	-	-	-	-	1	-	-	-	-	-	-
15	74896235	G	MPI	nsSNV	0.001	-	-	-	-	1	-	-	-	-	-	-
15	89320917	T	POLG	nsSNV	0.002	-	-	-	-	1	-	-	-	-	-	-
17	7482918	A	SLC35G6	nsSNV	0.005	-	-	-	-	1	-	-	-	-	-	-
17	36537558	T	PIGW	nsSNV	0.0005	-	-	-	-	1	-	-	-	-	-	-
18	50920565	A	ME2	nsSNV	-	-	-	-	1	1	1	-	-	-	-	-
20	34942942	T	GSS	nsSNV	-	-	-	-	-	1	-	-	-	-	-	-
22	37983654	C	SOX10	nsSNV	0.003	-	-	-	-	1	-	-	-	-	-	-

[a]Healthy subject.

1. Fatini C, Attanasio M, Porciani C, Sticchi E, Padeletti L, Lapini I, Abbate R, Gensini GF, and Pepe G, *AGT and ACE genes influence classic mitral valve prolapse predisposition in Marfan patients.* Int J Cardiol, 2008. **123**(3): p. 293-7.
2. Gentilini D, Oliveri A, Fazia T, Pini A, Marelli S, Bernardinelli L, and Di Blasio AM, *NGS analysis in Marfan syndrome spectrum: Combination of rare and common genetic variants to improve genotype-phenotype correlation analysis.* PLoS One, 2019. **14**(9): p. e0222506.
3. Lerner-Ellis JP, Aldubayan SH, Hernandez AL, Kelly MA, Stuenkel AJ, Walsh J, and Joshi VA, *The spectrum of FBN1, TGFβR1, TGFβR2 and ACTA2 variants in 594 individuals with suspected Marfan Syndrome, Loeys-Dietz Syndrome or Thoracic Aortic Aneurysms and Dissections (TAAD).* Mol Genet Metab, 2014. **112**(2): p. 171-6.
4. Ritelli M, Dordoni C, Venturini M, Chiarelli N, Quinzani S, et al., *Clinical and molecular characterization of 40 patients with classic Ehlers-Danlos syndrome: identification of 18 COL5A1 and 2 COL5A2 novel mutations.* Orphanet J Rare Dis, 2013. **8**: p. 58.
5. Gan Q, Liu Q, Hu X, and You C, *Collagen Type I Alpha 2 (COL1A2) Polymorphism Contributes to Intracranial Aneurysm Susceptibility: A Meta-Analysis.* Med Sci Monit, 2017. **23**: p. 3240-3246.
6. Wenstrup RJ, Florer JB, Willing MC, Giunta C, Steinmann B, Young F, Susic M, and Cole WG, *COL5A1 haploinsufficiency is a common molecular mechanism underlying the classical form of EDS.* Am J Hum Genet, 2000. **66**(6): p. 1766-76.
7. Li G, Yu J, Wang K, Wang B, Wang M, Zhang S, Qin S, and Yu Z, *Exome sequencing identified new mutations in a Marfan syndrome family.* Diagn Pathol, 2014. **9**: p. 25.
8. Li J, Lu C, Wu W, Liu Y, Wang R, Si N, Meng X, Zhang S, and Zhang X, *Application of next-generation sequencing to screen for pathogenic mutations in 123 unrelated Chinese patients with Marfan syndrome or a related disease.* Sci China Life Sci, 2019. **62**(12): p. 1630-1637.
9. Tsipouras P, Del Mastro R, Sarfarazi M, Lee B, Vitale E, et al., *Genetic linkage of the Marfan syndrome, ectopia lentis, and congenital contractural arachnodactyly to the fibrillin genes on chromosomes 15 and 5. The International Marfan Syndrome Collaborative Study.* N Engl J Med, 1992. **326**(14): p. 905-9.
10. Uyeda T, Takahashi T, Eto S, Sato T, Xu G, Kanezaki R, Toki T, Yonesaka S, and Ito E, *Three novel mutations of the fibrillin-1 gene and ten single nucleotide polymorphisms of the fibrillin-3 gene in Marfan syndrome patients.* J Hum Genet, 2004. **49**(8): p. 404-407.
11. Meester JA, Vandeweyer G, Pintelon I, Lammens M, Van Hoorick L, et al., *Loss-of-function mutations in the X-linked biglycan gene cause a severe syndromic form of thoracic aortic aneurysms and dissections.* Genet Med, 2017. **19**(4): p. 386-395.
12. Busnadiego O, Gorbenko Del Blanco D, González-Santamaría J, Habashi JP, Calderon JF, et al., *Elevated expression levels of lysyl oxidases protect against aortic aneurysm progression in Marfan syndrome.* J Mol Cell Cardiol, 2015. **85**: p. 48-57.
13. Loeys BL, Mortier G, and Dietz HC, *Bone lessons from Marfan syndrome and related disorders: fibrillin, TGF-B and BMP at the balance of too long and too short.* Pediatr Endocrinol Rev, 2013. **10 Suppl 2**: p. 417-23.
14. Morlino S, Alesi V, Calì F, Lepri FR, Secinaro A, et al., *LTBP2-related "Marfan-like" phenotype in two Roma/Gypsy subjects with the LTBP2 homozygous p.R299X variant.* Am J Med Genet A, 2019. **179**(1): p. 104-112.
15. Giusti B, Porciani MC, Brunelli T, Evangelisti L, Fedi S, et al., *Phenotypic variability of cardiovascular manifestations in Marfan Syndrome. Possible role of*

hyperhomocysteinemia and C677T MTHFR gene polymorphism. Eur Heart J, 2003. **24**(22): p. 2038-45.

16. Takeda N and Komuro I, *Genetic basis of hereditary thoracic aortic aneurysms and dissections.* J Cardiol, 2019. **74**(2): p. 136-143.
17. Cannaerts E, Kempers M, Maugeri A, Marcelis C, Gardeitchik T, et al., *Novel pathogenic SMAD2 variants in five families with arterial aneurysm and dissection: further delineation of the phenotype.* J Med Genet, 2019. **56**(4): p. 220-227.
18. Holm TM, Habashi JP, Doyle JJ, Bedja D, Chen Y, et al., *Noncanonical TGFβ signaling contributes to aortic aneurysm progression in Marfan syndrome mice.* Science, 2011. **332**(6027): p. 358-61.
